# Intraperitoneal Paclitaxel Treatment for Patients with Pancreatic Ductal Adenocarcinoma with Peritoneal Dissemination Provides a Survival Benefit

**DOI:** 10.3390/cancers14051354

**Published:** 2022-03-07

**Authors:** Tomohisa Yamamoto, Sohei Satoi, So Yamaki, Daisuke Hashimoto, Mitsuaki Ishida, Tsukasa Ikeura, Satoshi Hirooka, Yuki Matsui, Shogen Boku, Shinji Nakayama, Koh Nakamaru, Nobuhiro Shibata, Utae Katsushima, Mitsugu Sekimoto

**Affiliations:** 1 Department of Surgery, Kansai Medical University, Osaka 573-1010, Japan; yamamtom@hirakata.kmu.ac.jp (T.Y.); yamakis@hirakata.kmu.ac.jp (S.Y.); daisukeh007@gmail.com (D.H.); ss_largehill@yahoo.co.jp (S.H.); matsuiyk@hirakata.kmu.ac.jp (Y.M.); sekimotm@hirakata.kmu.ac.jp (M.S.); 2 Division of Surgical Oncology, University of Colorado Anschutz Medical Campus, Aurora, CO 80204, USA; 3 Department of Pathology and Clinical Laboratory, Kansai Medical University, Osaka 573-1010, Japan; mitsuaki.ishida@gmail.com; 4 The Third Department of Internal Medicine, Kansai Medical University, Osaka 573-1010, Japan; ikeurat@hirakata.kmu.ac.jp (T.I.); nakayams@takii.kmu.ac.jp (S.N.); nakamako@hirakata.kmu.ac.jp (K.N.); 5 Cancer Treatment Center, Kansai Medical University Hospital, Osaka 573-1010, Japan; shogen0820@gmail.com (S.B.); shibanob.kmu@gmail.com (N.S.); 6 Department of Physical Medicine and Rehabilitation, Kansai Medical University Hospital, Osaka 573-1010, Japan; poemblessing@yahoo.co.jp

**Keywords:** pancreatic cancer, peritoneal dissemination, intraperitoneal therapy, paclitaxel

## Abstract

**Simple Summary:**

Pancreatic ductal adenocarcinoma (PDAC) with peritoneal dissemination is a highly lethal disease. Recently, promising activity of intraperitoneal chemotherapy with paclitaxel (i.p.-PTX) has been observed in patients with peritoneal dissemination. We conducted a retrospective comparative study to evaluate the clinical efficacy of i.p.-PTX combined with systemic chemotherapy versus standard systemic chemotherapy in PDAC patients with peritoneal dissemination. The median survival time was 10.2 months for patients in the standard therapy group and 17.9 months in the i.p.-PTX group; the difference between groups was statistically significant (*p* = 0.006). We have performed surgical resection (defined as conversion surgery) to responders to treatment. Conversion surgery was planned for 26% in the i.p.-PTX group and 8% in the standard therapy group. The median survival time (27.4 months) from initial treatment in patients who underwent conversion surgery was significantly longer than that in patients who did not undergo conversion surgery (11.3 months, *p* < 0.0001). Implementation of the i.p.-PTX regimen may improve survival in patients with PDAC with peritoneal dissemination.

**Abstract:**

Background: Intraperitoneal chemotherapy using paclitaxel (i.p.-PTX) is expected to be a new therapeutic strategy for patients with pancreatic ductal adenocarcinoma (PDAC) and peritoneal dissemination. We evaluated the survival benefit of i.p.-PTX compared with standard systemic chemotherapy. Methods: Clinical data of 101 consecutive PDAC patients with peritoneal dissemination between 2007 and 2018 were analyzed. All patients were determined to have no other sites of distant organ metastasis to the lung, bone, or liver on contrast-enhanced CT imaging. Patients underwent staging laparoscopy or open laparotomy to confirm pathological evidence of peritoneal dissemination, and to exclude occult liver metastasis. Survival curves were estimated using the Kaplan–Meier method, and differences were compared using the log-rank test. Results: Forty-three patients were treated with i.p.-PTX (i.p.-PTX group) and forty-nine patients received standard systemic chemotherapy (Ctrl group). Nine patients did not receive any treatment (BSC group). The median survival time (MST) in the i.p.-PTX group was significantly longer than that in the Ctrl group (17.9 months vs. 10.2 months, *p* = 0.006). Negative peritoneal washing cytology was observed in 24 out of 43 patients in the i.p.-PTX group. The i.p.-PTX group tended to have a higher proportion of clinical responses than the Ctrl group (30% vs. 18%, *p* = 0.183). Conversion surgery was performed in 10 patients in the i.p.-PTX group and 2 patients in the Ctrl group after confirming disappearance of peritoneal dissemination with staging laparoscopy or open laparotomy (*p* = 0.005). The MST in patients who underwent surgical resection was significantly longer than that in patients who did not (27.4 months vs. 11.3 months; *p* < 0.0001). Conclusion: i.p.-PTX therapy provided improved survival in PDAC patients with peritoneal dissemination, and conversion surgery enhanced it in patients with favorable responses to chemotherapy. i.p.-PTX might become one of the treatment options to PDAC patients with peritoneal dissemination.

## 1. Introduction

Pancreatic ductal adenocarcinoma (PDAC) is a highly lethal disease with a close association between incidence and mortality within 1 year. Surgery with curative intent is recommended for the 15–20% of patients who present with resectable tumors. Fewer than 1 in 5 patients have early-stage disease amenable to potentially curative resection, and only 20% of those patients survive 5 years [1,2]. More than half of patients are diagnosed with metastatic disease and are not candidates for curative surgery. As a result, systemic chemotherapy is the only viable treatment option for most patients diagnosed with metastatic pancreatic cancer.

For years, the standard treatment for PDAC was single-agent gemcitabine [3]. However, 5-fluorouracil (5-FU), leucovorin, irinotecan, and oxaliplatin (FOLFIRINOX) significantly increased progression-free survival (PFS) and overall survival (OS) in the Accord 11 trial, establishing this regimen as a first-line therapeutic option [4]. Additionally, in 2013, the MPACT trial showed improved survival with gemcitabine plus nab-paclitaxel (GnP) vs. gemcitabine alone, introducing another valid front-line option [5]. However, the median survival time (MST) in these trials was still poor at less than 12 months.

One of the main metastatic pathways of PDAC is peritoneal dissemination, which is classified as either macroscopic, appearing as peritoneal deposits, or microscopic, presenting as cancer cells in ascites or in peritoneal lavage (CY^+^). The peritoneum is the second most common metastatic site following the liver, and peritoneal dissemination is present in 50% of patients with PDAC at the time of death [6,7]. Approximately 9% of PDAC patients already have established peritoneal dissemination at the time of diagnosis [8]. Peritoneal dissemination may be clinically divided into occult type diagnosed incidentally during open laparotomy or staging laparoscopy, and radiological detection type showing the presence of massive ascites, omental cake, intestinal obstruction, hydronephrosis [8,9], and concomitant organ metastases on imaging. The former is primarily asymptomatic, but 60–80% of patients have been reported to suffer from ascites within 1 year after initial treatment (MST: 7–10 months) [10,11]. Patients with the latter type suffer from various symptoms and cannot continue to receive systemic chemotherapy for long periods of time, resulting in a poor prognosis (MST: 2–4 months) [8,12,13]. Establishment of a standardized treatment approach for peritoneal dissemination is a critical issue in the management of PDAC.

Recently, promising activity of intraperitoneal chemotherapy with paclitaxel (i.p.-PTX) has been observed in select patients with peritoneal dissemination from various cancers. Peritoneal carcinomatosis of ovarian and gastrointestinal cancers can be treated with locoregional intraperitoneal chemotherapy and cytoreductive surgery with promising results [14,15]. Our previous trials of i.p.-PTX combined with S-1 and intravenous paclitaxel or GnP showed acceptable toxicity and favorable efficacy against peritoneal dissemination in patients with PDAC [16,17]. A preliminary report from our institution comparing clinical outcomes between intraperitoneal and systemic chemotherapy has been published: our retrospective study revealed that implementation of the S-1 plus intraveous/i.p.-PTX regimen (*n* = 20) was closely associated with prevention of ascites and higher resectability, resulting in improvement of OS in chemotherapy-naive patients with PDAC with peritoneal metastasis, relative to 29 patients who received standard systemic chemotherapy [13].

Herein, we conducted a retrospective comparative study in a single institution to evaluate the clinical efficacy of i.p.-PTX combined with systemic chemotherapy versus standard systemic chemotherapy in PDAC patients with peritoneal dissemination but without other distant organ metastasis on a large scale.

## 2. Methods

### 2.1. Patients

This was a retrospective analysis of data collected from Kansai Medical University Hospital for PDAC patients with peritoneal dissemination. The data from some patients have already been published in previous articles [18], and a pooled analysis contributed new data to this study.

### 2.2. Eligibility Criteria

Key inclusion criteria were: PDAC with histological or cytological diagnosis and peritoneal dissemination confirmed by staging laparoscopy or open laparotomy consisting of (i) presence of microscopic peritoneal dissemination during staging laparoscopy in patients with radiographically defined unresectable locally advanced PDAC, or (ii) presence of macroscopic peritoneal dissemination on staging laparoscopy or open laparotomy in patients with PDAC of any resectability status of primary tumor according to NCCN guidelines [19]. Key exclusion criteria were: presence of other sites of distant metastases excluding the ovaries, positive peritoneal cytology in patients without peritoneal deposits in otherwise resectable PDAC, and active concomitant malignancies.

### 2.3. Treatment

Clinical effectiveness of the i.p.-PTX treatment concomitant with S-1+i.v. PTX [16], gemcitabine+nab-PTX [17] or gemcitabine+S-1 (not published), as clinical trials has been evaluated since 2012. Except the duration of clinical trials, systemic chemotherapy has only been implemented in 49 patients as a control group during a study period from 2007 to 2018. Patients were categorized into three groups based on treatment. The i.p.-PTX group included 43 patients. When macroscopic or microscopic peritoneal dissemination was detected during staging laparoscopy or open laparotomy, a peritoneal access port was implanted in the lower abdomen. Patients received one of the following i.p.-PTX combination regimens [16,17]: (i) S-1 plus paclitaxel: S-1 was orally administered for 14 consecutive days, followed by 7 days of rest, and paclitaxel was administered intravenously and intraperitoneally on days 1 and 8 (*n* = 19); (ii) Gemcitabine plus S-1 plus paclitaxel: intravenous gemcitabine was administered on days 1 and 8 plus S-1 orally on days 1 through 14 of a 21-day cycle, and paclitaxel was administered intraperitoneally on days 1 and 8 (*n* = 7); (iii) GnP plus paclitaxel: nab-paclitaxel and gemcitabine were administered intravenously and paclitaxel was administered intraperitoneally on days 1, 8, and 15, followed by a 1-week rest, and paclitaxel was administered intraperitoneally on days 1, 8, and 15 (*n* = 17). 

The control group (Ctrl group) included 49 patients who received several treatments, including gemcitabine alone (*n* = 22), S-1 alone (*n* = 2), gemcitabine plus S-1 (*n* = 9), GnP (*n* = 10), modified FOLFIRINOX (*n* = 1), and chemoradiotherapy (*n* = 5) as standard systemic chemotherapy [20,21,22,23].

We have performed surgical resection (defined as conversion surgery) to responders to treatment. The following criteria were used: Eastern Cooperative Oncology Group performance status of 0 or 1, marked primary tumor shrinkage in patients with locally advanced PDAC, decreased (<150 U/mL) or normalization of tumor marker levels, washing cytology via peritoneal access port becoming negative (in two sequential tests), and disappearance of peritoneal deposits on staging laparoscopy. The decision to proceed to conversion surgery was based on an interval exceeding 8 months between initial treatment and surgical resection for confirming new emerging distant organ metastasis to the liver, lungs, or other organs.

The best supportive care group (BSC group) consisted of nine patients who did not receive chemo (radio)therapy due to poor performance status or refusal of chemotherapy.

### 2.4. Study Endpoints

The primary endpoint was OS, which was defined as the time from treatment initiation to death from any cause. Secondary endpoints were resection rate, clinical response rate, and changes in CA19-9 levels between groups, and time to peritoneal cytology becoming negative in the i.p.-PTX group. Objective tumor responses were classified according to the Response Evaluation Criteria in Solid Tumors (RECIST) guidelines, version 1.1 [24].

### 2.5. Data Collection

Clinical data were collected prospectively for all patients and included patient demographics, pathologic examination, peri-operative clinical information, and complications.

### 2.6. Statistical Analysis

The median follow-up period was 11.4 (range: 0.2–99.6) months; only three patients were alive at the time of analysis. For categorical variables, the chi-squared test was used to examine differences between groups; for numerical variables and nonparametric independent samples, the Mann–Whitney U test was used. Survival curves were calculated using the Kaplan–Meier method, and differences were compared using the log-rank test. Hazard ratios (HRs) with 95% confidence intervals (CIs) and two-sided *p*-values were reported. HRs in subgroups according to baseline characteristics and two-tailed 95%CIs were calculated using the Cox proportional hazards model. A *p*-value of less than 0.05 was considered statistically significant. All statistical analyses were performed using JMP Pro version 14.0 (SAS Institute, Cary, NC, USA).

### 2.7. Ethical Statement

The study was reviewed and approved (ref. No. 2020131) by the institutional review board of Kansai Medical University, Japan, and complied with the Strengthening the Reporting of Observational Studies in Epidemiology (STROBE) guidelines [25]. All procedures in this study were performed in accordance with the guidelines of the Declaration of Helsinki.

## 3. Results

### 3.1. Demographic and Clinical Data

**Figure 1:**
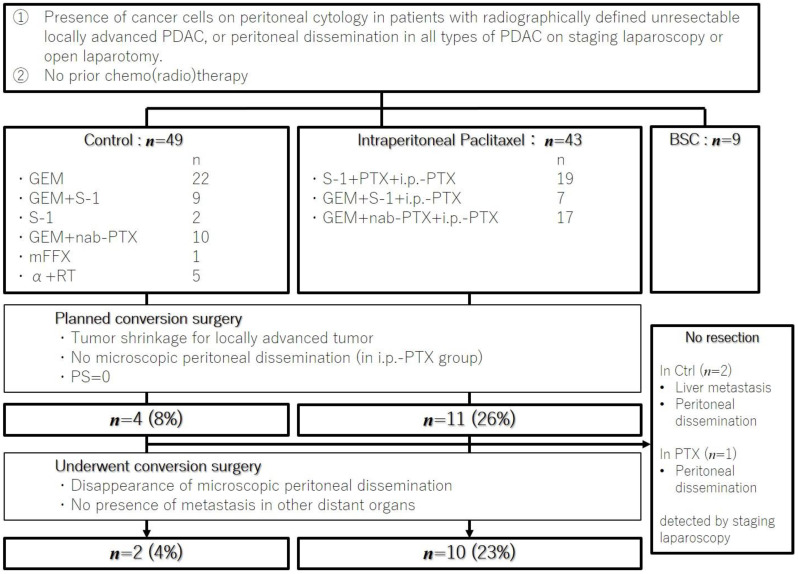
Flowchart of case selection. GEM, gemcitabine; nab-PTX, nab-paclitaxel; α, gemcitabine or S-1; RT, radiotherapy; mFFX, modified FOLFIRINOX; i.p.-PTX, intraperitoneal paclitaxel; PS, performance status; Ctrl, control; BSC, best supportive care.

Figure 1 illustrates the patient selection flow. A total of 101 eligible patients were included in the analysis; all patients were diagnosed between January 2007 and December 2018. The clinical characteristics of the i.p.-PTX and Ctrl groups were compared, and results are shown in Table 1. No significant differences between groups were observed, except for sex.

### 3.2. Survival Analysis

**Figure 2:**
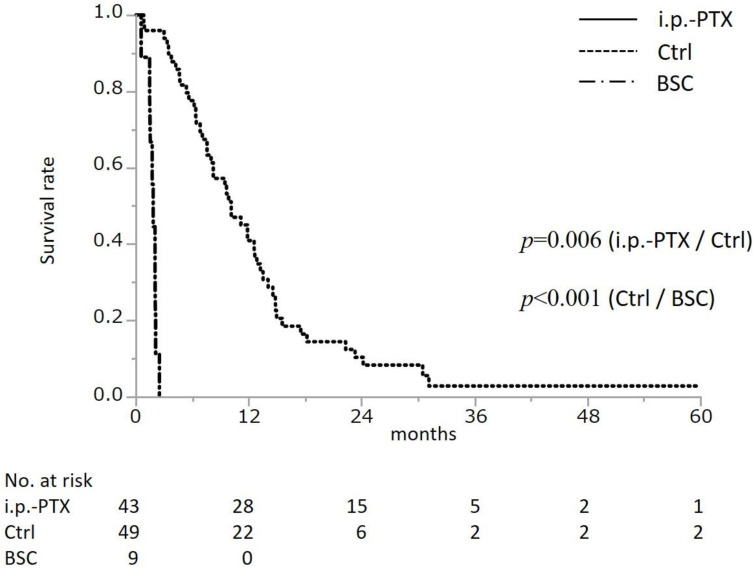
Overall survival following initial treatment. i.p.-PTX, intraperitoneal paclitaxel; Ctrl, control; BSC, best supportive care. The *p*-value was calculated by log-rank test. The median survival time was 17.9 months in the i.p.-PTX group (*n* = 43), 10.2 months in the Ctrl group (*n* = 49), and 1.9 months in the BSC group (*n* = 9).

As shown in Figure 2, the MST was 10.2 months for patients in the Ctrl group (95%CI, 7.6–12.7) and 17.9 months in the i.p.-PTX group (95%CI, 11.5–23.1; HR = 0.82; 95%CI, 0.36–0.85); the difference between groups was statistically significant (*p* = 0.006). In the Ctrl group, there were no significant differences in the MST between mono-chemotherapy (Gemcitabine, S-1, MST; 9.9 months) and combination chemotherapy (Gemcitabine+S-1, GnP, FOLFIRINOX, MST; 10.7 months) (*p* = 0.642). The MST in the BSC group (1.9 months, 95%CI, 0.6–2.1) was significantly shorter than that in the Ctrl group (*p* < 0.001).

Univariate analysis revealed that i.p.-PTX, clinical response (complete response [CR]/partial response [PR] rate according to RECIST criteria), and conversion surgery were significantly associated with survival (Table 2). Multivariate analysis revealed that only CR/PR rates and conversion surgery were significant independent predictive factors.

### 3.3. Conversion Surgery

**Figure 3:**
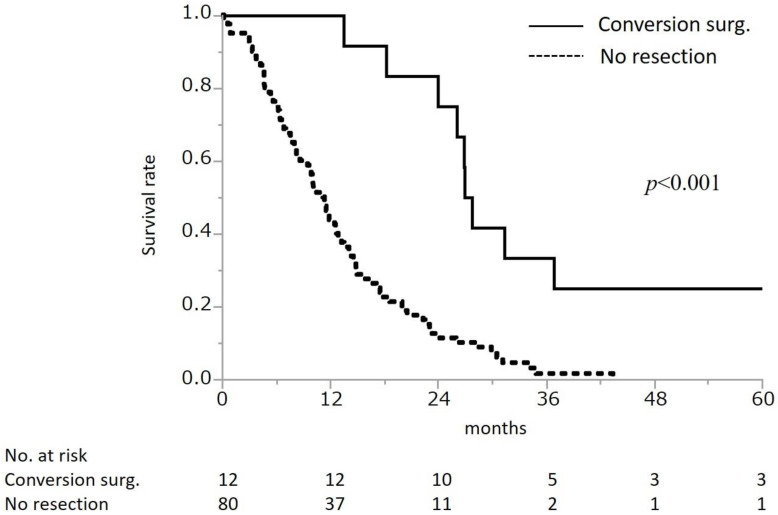
Overall survival from initial treatment by surgery status The *p*-value was calculated by log-rank test. The median survival time was 27.4 months in patients who underwent conversion surgery (*n* = 12), and 11.3 months in patients who did not undergo resection (*n* = 80).

**Figure 4:**
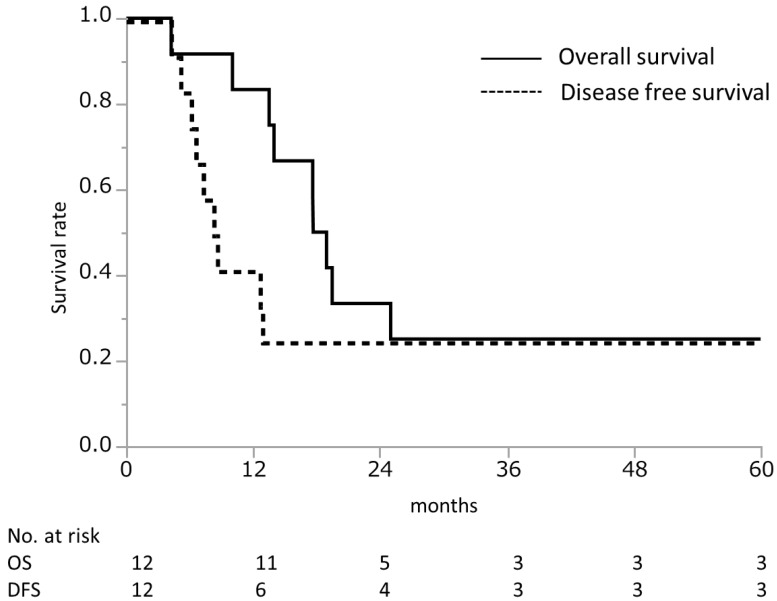
Overall survival and disease-free survival after conversion surgery. The median survival time was 18.3 months and disease-free survival was 8.4 months after conversion surgery in 12 patients.

The following criteria for surgical resection (defined as conversion surgery) were used: Eastern Cooperative Oncology Group performance status of 0 or 1, marked primary tumor shrinkage in patients with locally advanced PDAC, decreased (<150 U/mL) or normalization of tumor marker levels, washing cytology via peritoneal access port becoming negative (in two sequential tests), and disappearance of peritoneal deposits on staging laparoscopy. The decision to proceed to conversion surgery was based on an interval exceeding 8 months between initial treatment and surgical resection for confirming new emerging distant organ metastasis to the liver, lungs, or other organs. According to the criteria described above, conversion surgery was planned for 11 patients (26%) in the i.p.-PTX group and 4 patients (8%) in the Ctrl group. Liver or peritoneal metastasis by staging laparoscopy was detected in one patient in the i.p.-PTX group and two patients in the Ctrl group prior to conversion surgery (Figure 1). As a result, 12 patients underwent conversion surgery, with no hospital deaths (Supplementary Table S1). The MST (27.4 months; 95%CI, 18.2–89.2) from initial treatment in patients who underwent conversion surgery was significantly longer than the MST in patients who did not undergo conversion surgery (11.3 months; 95%CI, 8.3–12.9; HR = 0.24; 95%CI, 0.11–0.48; log-rank test, *p* < 0.0001) (Figure 3). The MST from conversion surgery was 18.3 months (95%CI, 10.0–82.9) and disease-free survival (DFS) was 8.4 months (95%CI, 5.1–82.9) (Figure 4). Adjuvant chemotherapy consisting of i.p.-PTX with S-1 was administered until confirmation of recurrence in the i.p.-PTX group or consisting of gemcitabine for 6 months in the Ctrl group. The 1-year DFS rate was 41.7%. Recurrence sites included local recurrences in three patients, the liver in one patient, and the peritoneum in four patients. Two patients without recurrence following conversion surgery survived beyond 5 years. 

**Figure 5:**
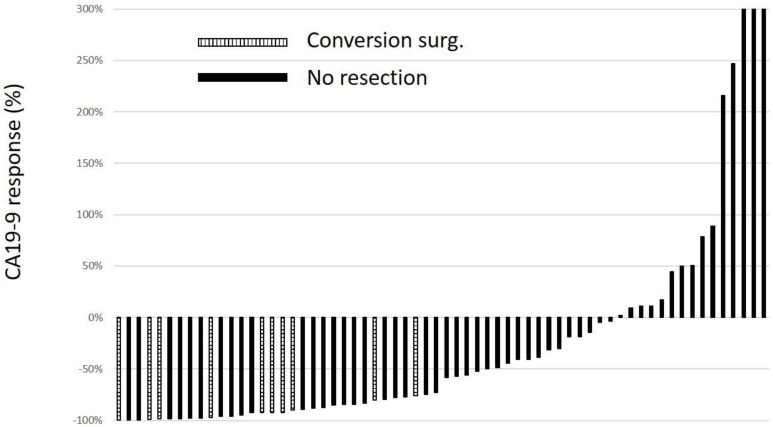
Waterfall plot of serum CA19-9 response from baseline for primary treatment. The data represent the rate of CA19-9 response, calculated as [(post treatment value/baseline value) − 1] (%). All patients were within normal CA19-9 levels of pretreatment; patients for whom CA19-9 levels were evaluated only once were excluded.

In both groups, pre-treatment CA19-9 levels ranged from 1.0 to 18,289 U/mL, and 17 patients had normal CA19-9 levels. When patients with normal CA19-9 levels were excluded, the median value of percent change in CA19-9 levels during first-line chemotherapy was −52.1% (range: −99.8% to 1072%) in 79 patients. A significant difference in the CA19-9 percent change between patients who underwent conversion surgery (−92.5%; range: −99.8% to −75.7%) and patients who did not undergo resection (−51.0%; range: −99.6 to 1071) was observed. Interestingly, CA19-9 levels decreased more than 75% from baseline among patients who underwent conversion surgery (Figure 5). 

**Figure 6:**
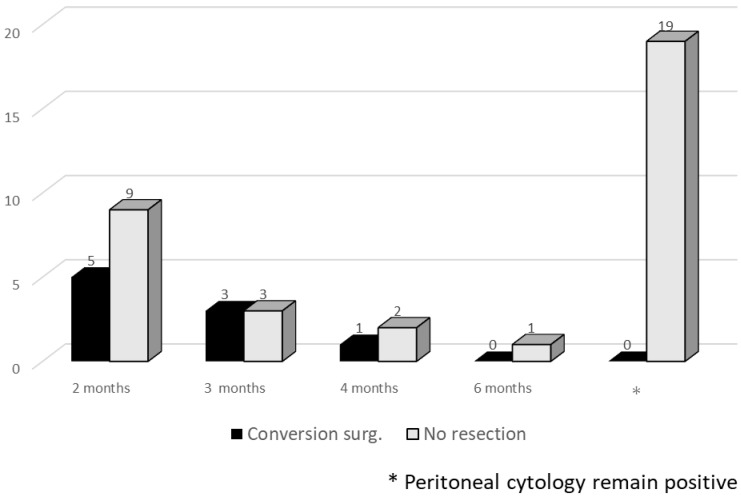
Time of peritoneal cytology becoming negative. Peritoneal washing cytology specimens were examined in i.p.-PTX group patients who had a peritoneal access port. Patients were categorized into five groups based on the time of peritoneal cytology becoming negative (<2 months, ≥2 months to <3 months, ≥3 months to <4 months, ≥4 months to <6 months, and peritoneal cytology positive).

Peritoneal washing cytology specimens were examined each month in patients in the i.p.-PTX group who had a peritoneal access port. Peritoneal washing cytology became negative in 24 of 43 patients (56%). Patients were categorized into five groups based on the time peritoneal cytology results becoming negative (<2 months: *n* = 14; ≥2 months to <3 months: *n* = 6; ≥3 months to <4 months: *n* = 3; ≥4 months to <6 months: *n* = 1; and peritoneal cytology remaining positive) (Figure 6). Peritoneal cytology results became negative within 4 months in all patients who underwent conversion surgery. 

Table 3 clearly reveals that i.p.-PTX therapy, CA19-9 normalization, and CR or PR rate by RECIST were significant predictive factors for conversion surgery.

## 4. Discussion

Treatment of PDAC with peritoneal dissemination usually requires systemic chemotherapy to delay tumor progression and increase survival time; nevertheless, the outcomes of such non-surgical palliative treatment are not satisfactory. Current standard first-line regimens for patients with metastatic disease include modified FOLFIRINOX [4,21] or GnP [5,12]; however, median OS remains poor, ranging from 8.5–11.1 months. Despite these improvements, the MST of metastatic pancreatic cancer is still less than 1 year. The efficacy of anticancer drugs generally depends on the concentration and duration of tumor exposure to chemotherapeutic agents. Intraperitoneal chemotherapy enables intraperitoneal tumors to be exposed to high concentrations of chemotherapeutic agents to directly contact the target tumor, without increasing blood concentrations to toxic levels. Paclitaxel is an appropriate agent for intraperitoneal administration because of slow absorption through the lymphatic system, due to its large molecular weight and fat solubility [26].

Intraperitoneal administration of paclitaxel was developed to enhance antitumor activity against peritoneal dissemination by maintaining a high drug concentration in the peritoneal cavity over a long period, and its clinical efficacy has been verified by several convincing clinical trials in ovarian, gastric, and pancreatic cancers with peritoneal dissemination [14,15,16,17,18]. Ishigami et al. reported that i.p.-PTX with systemic chemotherapy had promising clinical efficacy in gastric cancer [15]. We also previously reported promising clinical efficacy and acceptable tolerability of i.p.-PTX therapy in PDAC patients with peritoneal dissemination [16,17]. In our multicenter phase II study in 33 PDAC patients with peritoneal dissemination, our intravenous/i.p.-PTX plus S-1 combination regimen yielded a response rate of 36% and MST of 16.3 (range: 11.47–22.57) months [16]. In addition, in a second trial that evaluated intravenous gemcitabine, intravenous nab-paclitaxel, and i.p.-PTX in 46 patients, a response rate of 48% and MST of 14.5 (range: 11.5–19.2) months were observed [12]. Considering these results from two phase II studies, a clinical practice guideline for PDAC with peritoneal dissemination in Japan stated that intraperitoneal chemotherapy is weakly recommended in patients with peritoneal dissemination who do not have a large amount of ascites (off-label use) [27]. 

Few publications comparing clinical outcomes in PDAC patients with peritoneal dissemination who received i.p.-PTX therapy and those who receive standard systemic chemotherapy exist. In the current study, clinical outcomes and follow-up data from 101 patients represented a single-center cohort of PDAC patients with peritoneal dissemination. The MST was significantly prolonged, with an increase of 7.7 months in the i.p.-PTX group (17.9 months) compared with the Ctrl group (10.2 months). Considering that patients with peritoneal dissemination generally have a particularly poor prognosis, the present survival results are encouraging.

Surgical resection has provided the only chance for cure in patients with PDAC. According to the international guidelines for the treatment of PDAC and widespread clinical practice, surgical resection of metastatic disease is not recommended and, therefore, not routinely performed in clinical practice [19,28]. In this context, peritoneal dissemination has been considered a lethal disease with no curative surgical options. However, recent advances in chemotherapy may provide more opportunities for potentially curative resection in carefully selected patients, including those with metastatic disease [29,30,31,32,33,34]. In the current study, effective elimination of peritoneal deposits and intraperitoneal free cancer cells allowed us to perform conversion surgery in select patients. The rate of conversion surgery was 23% (10/43) in the i.p.-PTX group, which was significantly higher than the rate of 4% (2/49) in the Ctrl group (*p* = 0.005). Additionally, the MST in patients who underwent conversion surgery was significantly longer than that in patients who did not. Yamada et al. reported that conversion surgery after intraperitoneal treatment resulted in promising clinical efficacy with acceptable tolerability in patients with PDAC with peritoneal dissemination [33]. A clinical practice guideline in Japan also provided a weak recommendation that conversion surgery should be performed in patients whose peritoneal dissemination becomes undetectable macroscopically and microscopically [26]. A high proportion of patients were eligible for conversion surgery because i.p.-PTX therapy contributed to high response rates against macroscopic and microscopic peritoneal metastases, as well as primary tumors. It should be highlighted that two patients who underwent conversion surgery survived for longer than 5 years. Conversion surgery for PDAC patients with peritoneal dissemination can be effective for improving clinical outcomes, and i.p.-PTX may increase the chance of conversion surgery. 

The median DFS was only 8.4 months in patients who underwent conversion surgery. Sites of recurrence included local sites (*n* = 3), the liver (*n* = 1), and the peritoneum (*n* = 4). Among the eight patients with disease recurrence, six patients experienced an early recurrence within 1 year of conversion surgery. These patients received minimal benefit from undergoing conversion surgery of the primary tumor. Unfortunately, we could not identify risk factors for early recurrence due to the limited number of patients. Large cohort studies are needed to identify the optimal criteria for conversion surgery to improve postoperative outcomes.

CA19-9 is the most common and important tumor marker used in PDAC patients. Although baseline CA19-9 values have been shown to be associated with survival in patients with advanced, unresectable PDAC, conflicting evidence exists regarding the predictive value of peri-treatment CA19-9 in patients with unresectable PDAC treated with radiotherapy or chemotherapy [35,36]. In the current study, CA19-9 was evaluated at baseline and monthly after beginning treatment, and we calculated percent changes. CA19-9, which was abnormal at baseline in patients who underwent conversion surgery, decreased to more than 75% of baseline values and <70 U/mL. The role of serum CA19-9 may, thus, allow for decision-making that is better tailored to the patient’s biologic response and offer improved outcomes.

Cytological examination of peritoneal washing is considered as the gold standard for assessing the presence of free cancer cells in the peritoneal cavity. Ariake et al. reported that it was useful to evaluate washing cytology using peritoneal access ports after chemotherapy to determine the indication for surgical resection for PDAC patients with positive peritoneal cytology [37]. We consider that peritoneal washing cytology using peritoneal access ports is necessary for evaluating disease control during chemotherapy in patients with peritoneal dissemination. We have evaluated the timing of washing cytology becoming negative in patients with peritoneal access ports (i.p.-PTX group). Patients who underwent conversion surgery achieved negative conversion of their cytology status rapidly. The timing to convert washing cytology to negative may be useful for evaluating response of treatment and predicting conversion surgery outcomes.

This study has several limitations. First, this retrospective (non-randomized trial design), single-center study had a limited number of patients, leading to selection bias. i.p.-PTX treatment did not demonstrate a statistically significant correlation with survival in multivariate analysis. Our findings should stimulate further inquiry into how to initially manage unresectable PDAC with peritoneal dissemination. Second, conversion surgery was performed in a small number of patients who responded to chemotherapy. It is difficult to determine whether this is the result of including patients with favorable tumor biology or if conversion surgery may provide an actual survival advantage. Third, in the Ctrl group, various chemotherapy regimens were used, and few patients received recently introduced regimens, such as FOLFIRINOX or GnP. Clinical outcomes between i.p.-PTX combined with systemic chemotherapy and FOLOFIRINOX or GnP should be compared in the future.

In conclusion, i.p.-PTX therapy yielded promising clinical efficacy. Implementation of the i.p.-PTX regimen may improve survival in patients with PDAC with peritoneal dissemination because of the high proportion which performed conversion surgery. Currently, we are conducting a phase III randomized controlled trial to compare OS between S-1 plus intravenous/i.p.-PTX and GnP (UMIN000027229/jRCTs051180199).

## Figures and Tables

**Table 1:** Patient characteristics.

Variables	Control (*n* = 49)	i.p.-PTX (*n* = 43)	BSC (*n* = 9)	*p*-Value (Ctrl vs. i.p.-PTX)
Pre-treatment factors				
Gender, Male: Female, *n* (%)	34 (69): 15 (31)	16 (35): 28 (65)	4 (44): 5 (56)	0.001
Age, median (range), years	66 (41–85)	69 (42–81)	71 (65–75)	0.222
Performance status, 0:1:2, *n* (%)	43 (88): 6 (12): 0 (0)	35 (81): 8 (19): 0 (0)	3 (33): 2 (22): 4 (44)	0.397
Charlson Comorbidity Index, 2:3:4:5:6, *n* (%)	32(65): 32 (4): 13 (27): 1 (2): 1 (2)	28 (65): 3 (7): 12 (28): 0 (0): 0 (0)	7 (78): 2 (22): 0 (0): 0 (0): 0 (0)	0.577
Primary tumor site, Ph: Pbt, *n* (%)	22 (45): 27 (55)	13 (30): 30 (70)	3 (33): 6 (67)	0.147
Radiological tumor size, median (range), mm	41 (10–91)	43 (15–105)	50 (33–88)	0.891
NCCN resectability status of primary tumor, R:BR:UR, *n* (%)	11 (23): 8 (16): 30 (61)	10 (23): 5 (12): 28 (65)	0 (0): 8 (89): 1 (11)	0.809
Peritoneal nodule, *n* (%)	32 (65)	27 (63)	6 (67)	0.802
CA19-9, median (range), U/mL	215 (1.9–18289)	462 (1–8083)	2430 (118–18977)	0.153
Systemic chemotherapy regimen, *n*				
GEM	22	NA	NA	
GEM + S-1	9	7	NA	
S-1	2	NA	NA	
GEM + nab-PTX	10	17	NA	
mFFX	1	NA	NA	
S-1 + PTX	NA	19	NA	
α+ RT	5	NA	NA	
Post-treatment factors				
Normalization of CA19-9, *n* (%)	19 (38)	18 (42)	NA	0.763
Duration of primary treatment, median (range), months	6.8 (0.3–53.0)	7.6 (0.3- 24.6)	NA	0.583
Response, CR: PR: SD: PD, *n* (%)	1 (2): 8 (16): 33 (67): 7 (14)	0 (0): 13 (30): 22 (51): 8 (19)	NA	0.214
Proportion of conversion surgery, *n* (%)	2 (4)	10 (23)	NA	0.005

Ph, pancreas head; Pbt, pancreas body and tail; NCCN, National Comprehensive Cancer Network; R, resectable; BR, borderline resectable; UR, unresectable; CR, complete response; PR, partial response; SD, stable disease; PD, progressive disease; NA, not available; GEM, gemcitabine; nab-PTX, nab-paclitaxel; α, gemcitabine or S-1; RT, radiotherapy; mFFX, modified FOLFIRINOX; i.p.-PTX, intraperitoneal paclitaxel; Ctrl, control; BSC, best supportive care.

**Table 2:** Prognostic factors.

Variables	Univariate Analysis		Multivariate Analysis	
	HR (95%CI)	*p*-Value	HR (95%CI)	*p*-Value
Gender (male:female)	1.071 (0.704–1.634)	0.747		
Age, years (<70:≥70)	1.099 (0.721–1.691)	0.662		
Primary tumor site (Ph:Pbt)	1.019 (0.657–1.559)	0.929		
Radiological tumor size, mm (<40:≥40)	0.911 (0.588–1.392)	0.669		
NCCN resectability status of primary tumor (R/BR:UR)	1.034 (0.673–1.616)	0.881		
Peritoneal nodule (−:+)	0.881 (0.566–1.349)	0.563		
Pre-treatment CA19-9, U/mL (<300:≥300)	0.946 (0.614–1.457)	0.802		
Primary treatment (Ctrl:i.p.-PTX)	0.548 (0.355–0.843)	0.006	0.681 (0.438–1.051)	0.082
Response (CR/PR:SD/PD)	0.306 (0.171–0.520)	0.001	0.491 (0.250–0.895)	0.019
Conversion surgery (−:+)	0.246 (0.112–0.475)	<0.001	0.374 (0.178–0.707)	0.002

Ph, pancreas head; Pbt, pancreas body and tail; NCCN, National Comprehensive Cancer Network; R, resectable; BR, borderline resectable; UR, unresectable; i.p.-PTX, intraperitoneal paclitaxel; CR, complete response; PR, partial response; SD, stable disease; PD, progressive disease; HR, hazard ratio; CI, confidence interval.

**Table 3:** Univariate and multivariate analysis to predict conversion surgery.

Variables	Univariate Analysis		Multivariate Analysis	
	Odds Ratio (95%CI)	*p*-Value	Odds Ratio (95%CI)	*p*-Value
Pre-treatment factor				
Gender (male:female)	1.162 (0.337–4.012)	0.808		
Age, years (<70:≥70)	2.848 (0.098–11.401)	0.098		
Primary tumor site (Ph:Pbt)	1.190 (0.327–4.065)	0.783		
Radiological tumor size, mm (<40:≥40)	1.071 (0.295–3.651)	0.913		
NCCN resectability status of primary tumor (R/BR:UR)	1.031 (0.147–4.524)	0.971		
Peritoneal nodule (−:+)	1.962 (0.565–6.843)	0.282		
CA19-9, U/mL (<300:≥300)	1.472 (0.434–5.340)	0.535		
Post-treatment factor				
Normalization of CA19-9 (−:+)	9.815 (2.378–66.86)	0.001	7.537 (1.068–86.004)	0.043
Primary treatment (Ctrl: i.p.-PTX)	7.121 (1.735–48.33)	0.005	12.32 (1.831–142.92)	0.008
Response (CR/PR:SD/PD)	69.00 (11.76–1326.3)	0.001	62.47 (8.651–1381.3)	0.001

Ph, pancreas head; Pbt, pancreas body and tail; NCCN, National Comprehensive Cancer Network; R, resectable; BR, borderline resectable; UR, unresectable; Ctrl, control; i.p.-PTX, intraperitoneal paclitaxel; CR, complete response; PR, partial response; SD, stable disease; PD, progressive disease.

## Data Availability

The data presented in this study are available in this article (and supplementary material).

## Supplementary Materials

The following supporting information can be downloaded at: https://www.mdpi.com/article/10.3390/cancers14051354/s1, Table S1: Clinical characteristics of patients who underwent conversion surgery.
