# Supplementary material for: Intraperitoneal Paclitaxel Treatment for Patients with Pancreatic Ductal Adenocarcinoma with Peritoneal Dissemination Provides a Survival Benefit
Source: Cancers (Basel). 2022 Mar 7;14(5):1354. doi: 10.3390/cancers14051354 (PMC8909716; doi:10.3390/cancers14051354)
Supplement: Supplementary file 1 [file cancers-14-01354-s001.zip › cancers-1584249-supplementary.pdf]

cancers

MDPI

Supplementary Table.

Table S1. Clinical characteristics of patients who underwent conversion surgery.

No.	Gender	Age	Primary tumor site /NCCN resectability status	Radiological tumor size	Baseline CA19-9	Peritoneal nodule	Regimen	RECIST	minimum CA19-9	An interval since initial treatment	Operation	T	N	stage	R status	Evans	Recurrent site	DFS	OS since CS	Status
	M/F	y.o.		mm	U/ml	- / +			U/ml	months								months	months	Alive/Dead
1	M	64	Ph: UR	91	58	-	GEM+S-1 +RT	PR	14.1	8.2	PD	1b	0	1A	R0	III	Peritoneum	6.6	10.0	Dead
2	F	73	Ph: UR	27	1597	-	S-1+RT	CR	21.9	6.3	TP+PV+CHA	1a	0	1A	R0	III	none	82.9	82.9	Dead
3	F	60	Pbt: UR	35	130	-	S-1+PTX+i.p.-PTX	PR	26	8.8	DP-CAR+PV	2	1	2B	R1	IIa	Local	7.3	18.9	Dead
4	M	69	Pbt: UR	43	913	+	S-1+PTX+i.p.-PTX	PR	69.3	8.4	DP	2	1	2B	R0	IIa	Liver	5.1	17.7	Dead
5	M	81	Pbt: R	34	636	+	S-1+PTX+i.p.-PTX	PR	34.1	12.8	DP	1a	0	1A	R0	III	none	/	86.8	Alive
6	M	75	Pbt: UR	44	598	+	S-1+PTX+i.p.-PTX	PR	62.5	10.5	DP	2	1	2B	R0	IIa	Local	8.3	13.5	Dead
7	F	50	Pbt: UR	25	418	+	S-1+PTX+i.p.-PTX	PR	11.6	11.9	DP-CAR+PV	2	1	2B	R0	IIb	Peritoneum	8.6	25.0	Dead
8	F	74	Ph: UR	41	1778	-	S-1+PTX+i.p.-PTX	PR	13	13.0	TP+PV	1c	0	1A	R0	IIb	Peritoneum	12.6	14.0	Dead
9	F	73	Pbt: R	74	9.1	+	GEM+S-1+i.p.-PTX	PR	8.2	7.4	DP	3	1	2B	R0	IIa	Peritoneum	6.1	19.5	Dead
10	F	72	Pbt: UR	46	171.8	-	GEM+nab-PTX+i.p.-PTX	PR	13.3	9.3	PD+PV	1c	0	1A	R0	IIb	none	4.2	4.2	Dead
11	M	73	Ph: BR	52	266	+	GEM+nab-PTX+i.p.-PTX	PR	26.6	9.7	PD+PV	1b	0	1A	R0	III	none	/	30.2	Alive
12	F	74	Ph: UR	27	2957	-	GEM+nab-PTX+i.p.-PTX	PR	5	13.7	PD+PV	3	0	2A	R0	II a	Local	12.9	17.6	Dead

M, male; F, female; Ph, pancreas head; Pbt, pancreas body and tail; R, resectable; BR, borderline resectable; UR, unresectable; GEM, gemcitabine; nab-PTX, nab-paclitaxel; RT, radiotherapy; i.p.-PTX, intraperitoneal paclitaxel; CR, complete response; PR, partial response; PD, pancreaticoduodenectomy; DP, distal pancreatectomy; DP-CAR, distal pancreatectomy with en-bloc celiac axis resection; TP, total pancreatectomy; CHA, common hepatic artery resection; PV, portal vein resection.
